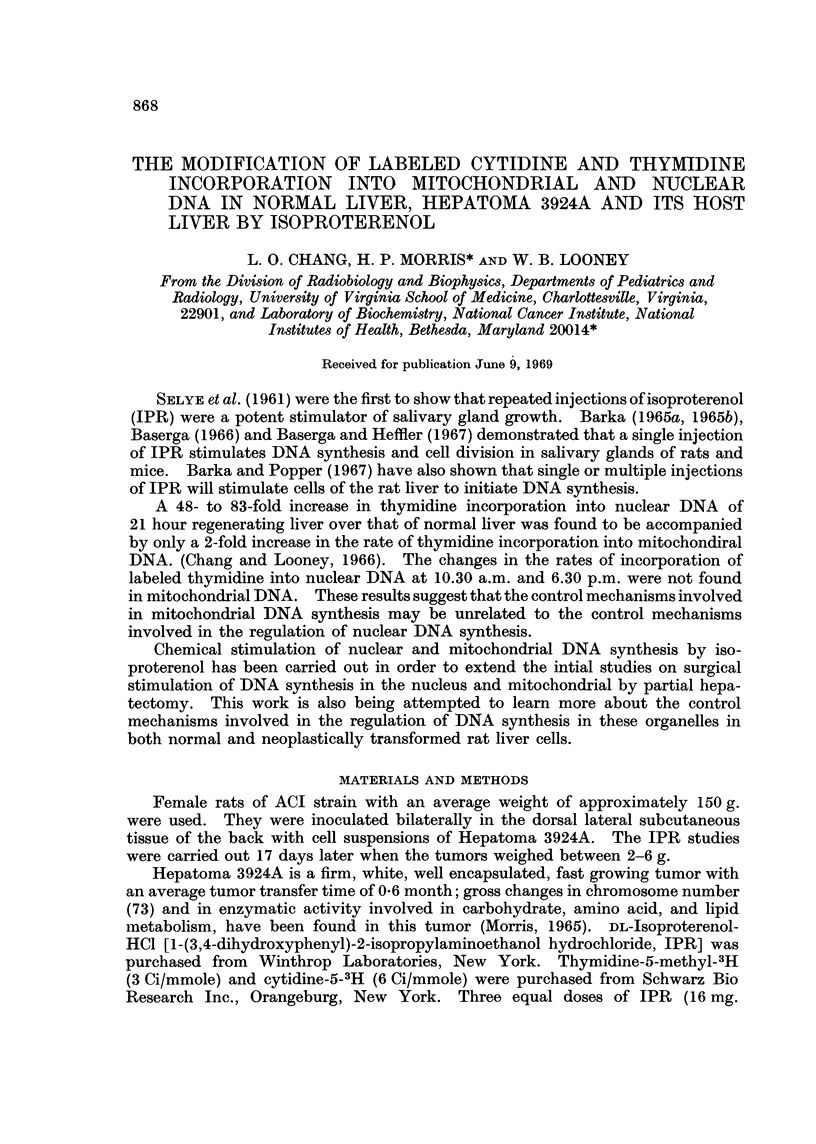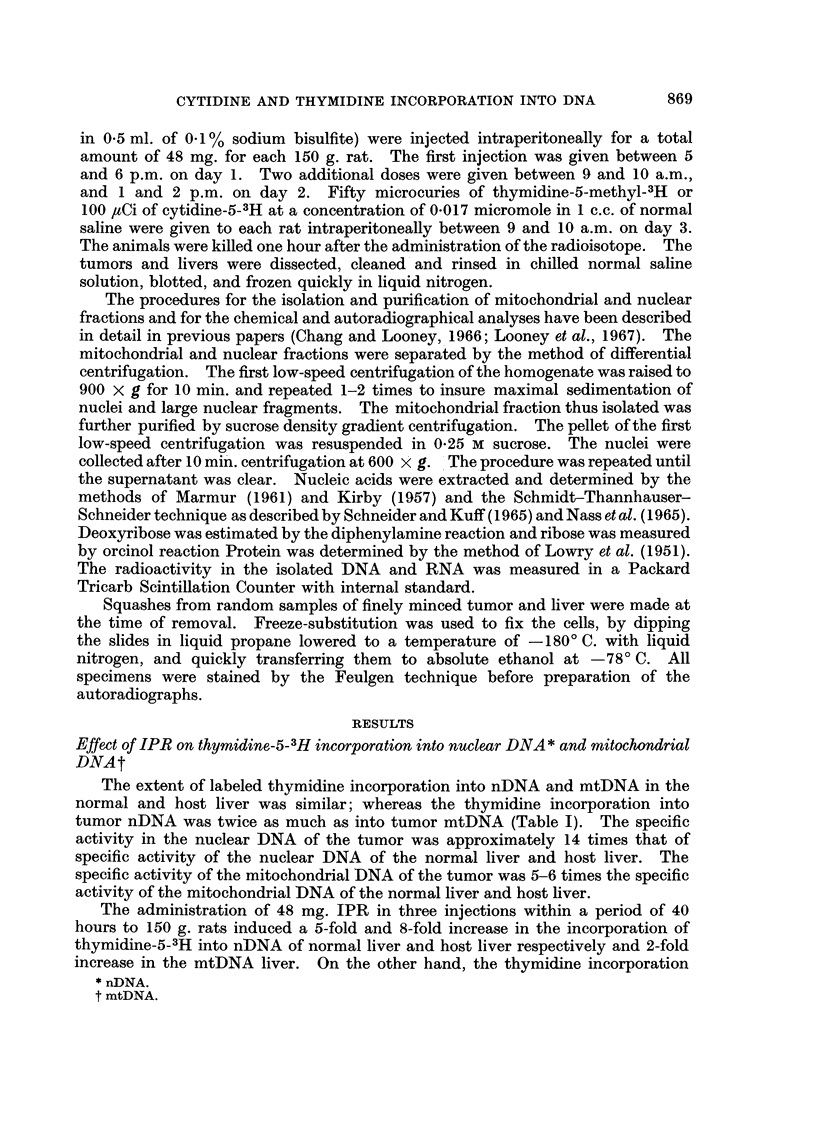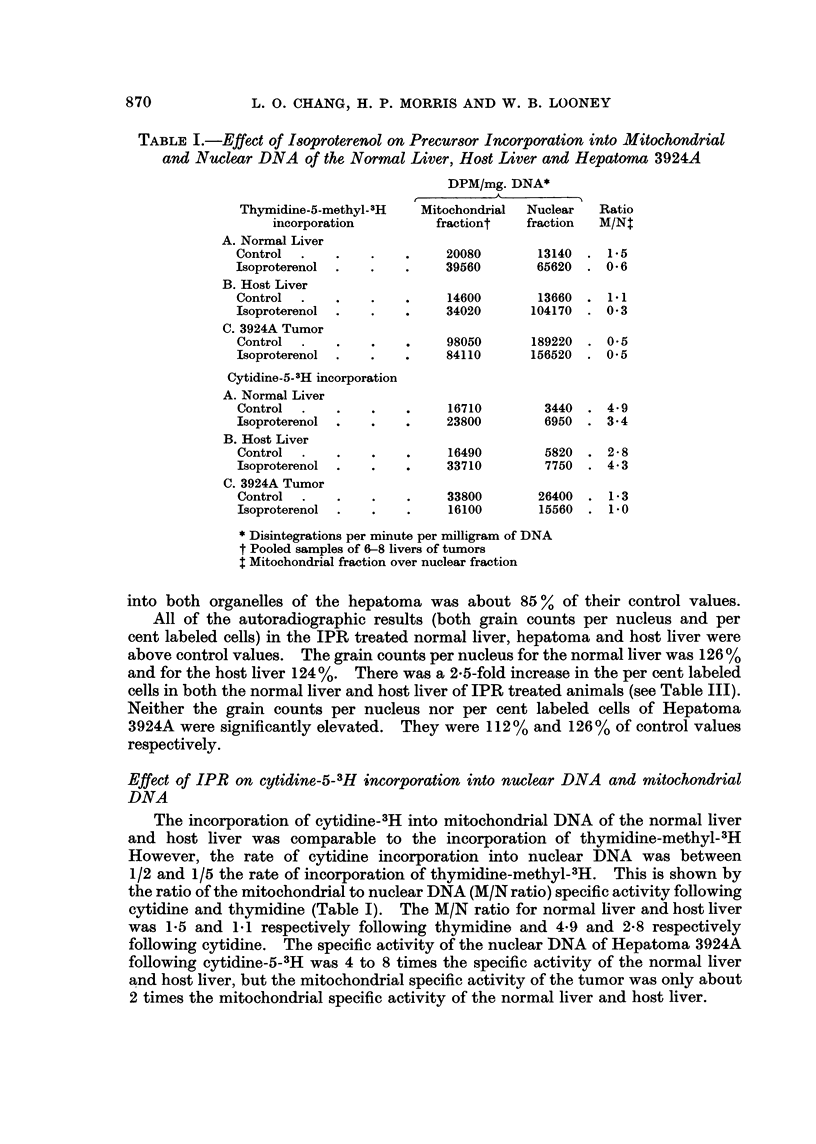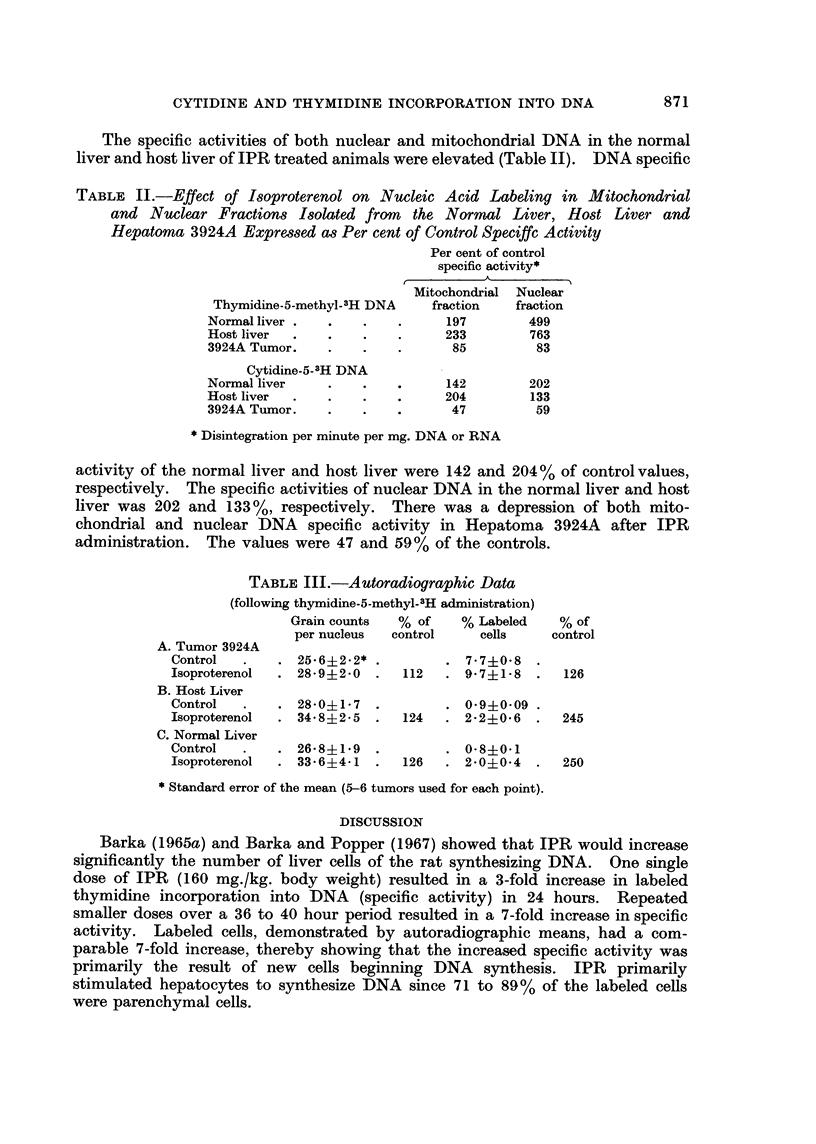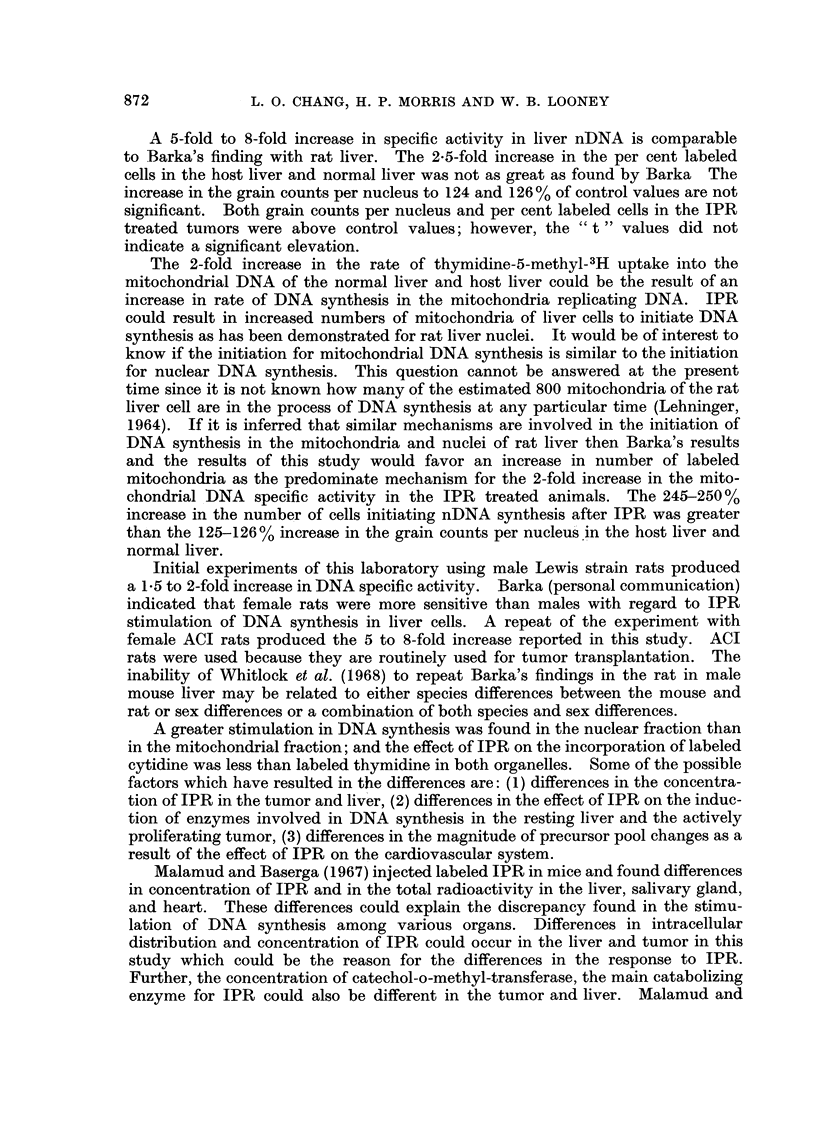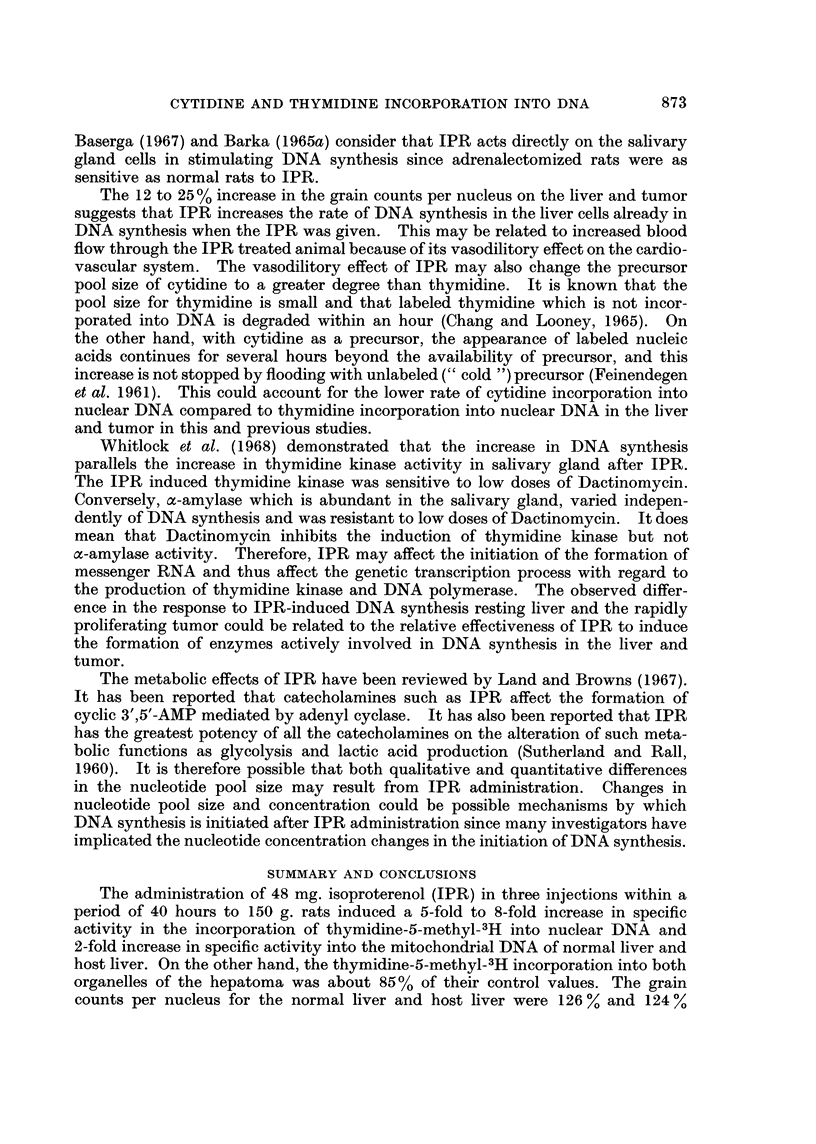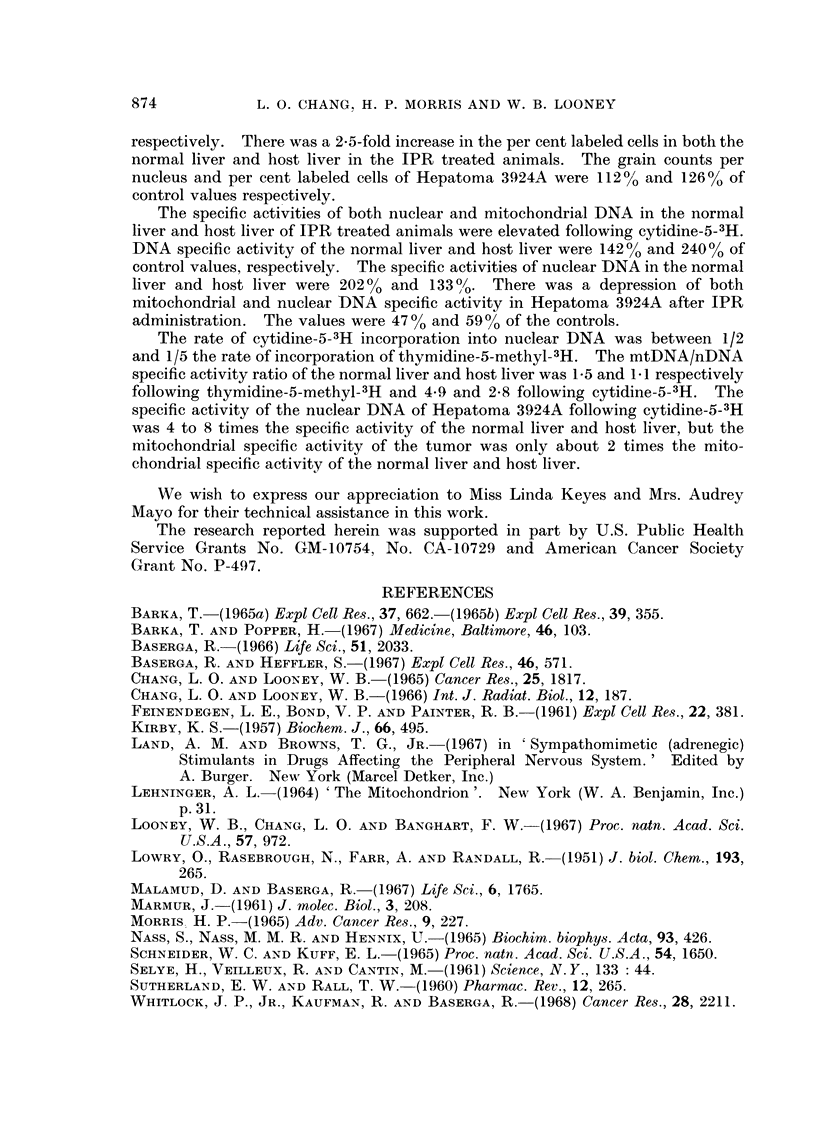# The modification of labeled cytidine and thymidine incorporation into mitochondrial and nuclear DNA in normal liver, hepatoma 3924A and its host liver by isoproterenol.

**DOI:** 10.1038/bjc.1969.107

**Published:** 1969-12

**Authors:** L. O. Chang, H. P. Morris, W. B. Looney


### THE MODIFICATION OF LABELED CYTIDINE AND THYMIDINE INCORPORATION INTO MITOCHONDRIAL AND NUCLEAR DNA IN NORMAL LIVER, HEPATOMA 3924A AND ITS HOST LIVER BY ISOPROTERENOL

L. O. CHANG, H. P. MORRIS* AND W. B. LOONEY

*From the Division of Radiobiology and Biophysics, Departments of Pediatrics and Radiology, University of Virginia School of Medicine, Charlottesville, Virginia,* 22901, *and Laboratory of Biochemistry, National Cancer Institute, National Institutes of Health, Bethesda, Maryland* 20014*

Received for publication June 9, 1969

SELYE *et al.* (1961) were the first to show that repeated injections of isoproterenol (IPR) were a potent stimulator of salivary gland growth. Barka (1965*a*, 1965*b*), Baserga (1966) and Baserga and Heffler (1967) demonstrated that a single injection of IPR stimulates DNA synthesis and cell division in salivary glands of rats and mice. Barka and Popper (1967) have also shown that single or multiple injections of IPR will stimulate cells of the rat liver to initiate DNA synthesis.

A 48- to 83-fold increase in thymidine incorporation into nuclear DNA of 21 hour regenerating liver over that of normal liver was found to be accompanied by only a 2-fold increase in the rate of thymidine incorporation into mitochondiral DNA. (Chang and Looney, 1966). The changes in the rates of incorporation of labeled thymidine into nuclear DNA at 10.30 a.m. and 6.30 p.m. were not found in mitochondrial DNA. These results suggest that the control mechanisms involved in mitochondrial DNA synthesis may be unrelated to the control mechanisms involved in the regulation of nuclear DNA synthesis.

Chemical stimulation of nuclear and mitochondrial DNA synthesis by isoproterenol has been carried out in order to extend the intial studies on surgical stimulation of DNA synthesis in the nucleus and mitochondrial by partial hepatectomy. This work is also being attempted to learn more about the control mechanisms involved in the regulation of DNA synthesis in these organelles in both normal and neoplastically transformed rat liver cells.

### MATERIALS AND METHODS

Female rats of ACI strain with an average weight of approximately 150 g. were used. They were inoculated bilaterally in the dorsal lateral subcutaneous tissue of the back with cell suspensions of Hepatoma 3924A. The IPR studies were carried out 17 days later when the tumors weighed between 2–6 g.

Hepatoma 3924A is a firm, white, well encapsulated, fast growing tumor with an average tumor transfer time of 0·6 month; gross changes in chromosome number (73) and in enzymatic activity involved in carbohydrate, amino acid, and lipid metabolism, have been found in this tumor (Morris, 1965). DL-Isoproterenol-HCl [1-(3,4-dihydroxyphenyl)-2-isopropylaminoethanol hydrochloride, IPR] was purchased from Winthrop Laboratories, New York. Thymidine-5-methyl-$^3$H (3 Ci/mmole) and cytidine-5-$^3$H (6 Ci/mmole) were purchased from Schwarz Bio Research Inc., Orangeburg, New York. Three equal doses of IPR (16 mg.

in 0·5 ml. of 0·1% sodium bisulfite) were injected intraperitoneally for a total amount of 48 mg. for each 150 g. rat. The first injection was given between 5 and 6 p.m. on day 1. Two additional doses were given between 9 and 10 a.m., and 1 and 2 p.m. on day 2. Fifty microcuries of thymidine-5-methyl-$^3$H or 100 $\mu$Ci of cytidine-5-$^3$H at a concentration of 0·017 micromole in 1 c.c. of normal saline were given to each rat intraperitoneally between 9 and 10 a.m. on day 3. The animals were killed one hour after the administration of the radioisotope. The tumors and livers were dissected, cleaned and rinsed in chilled normal saline solution, blotted, and frozen quickly in liquid nitrogen.

The procedures for the isolation and purification of mitochondrial and nuclear fractions and for the chemical and autoradiographical analyses have been described in detail in previous papers (Chang and Looney, 1966; Looney *et al.*, 1967). The mitochondrial and nuclear fractions were separated by the method of differential centrifugation. The first low-speed centrifugation of the homogenate was raised to 900 $\times$ *g* for 10 min. and repeated 1–2 times to insure maximal sedimentation of nuclei and large nuclear fragments. The mitochondrial fraction thus isolated was further purified by sucrose density gradient centrifugation. The pellet of the first low-speed centrifugation was resuspended in 0·25 M sucrose. The nuclei were collected after 10 min. centrifugation at 600 $\times$ *g*. The procedure was repeated until the supernatant was clear. Nucleic acids were extracted and determined by the methods of Marmur (1961) and Kirby (1957) and the Schmidt–Thannhauser–Schneider technique as described by Schneider and Kuff (1965) and Nass *et al.* (1965). Deoxyribose was estimated by the diphenylamine reaction and ribose was measured by orcinol reaction Protein was determined by the method of Lowry *et al.* (1951). The radioactivity in the isolated DNA and RNA was measured in a Packard Tricarb Scintillation Counter with internal standard.

Squashes from random samples of finely minced tumor and liver were made at the time of removal. Freeze-substitution was used to fix the cells, by dipping the slides in liquid propane lowered to a temperature of −180° C. with liquid nitrogen, and quickly transferring them to absolute ethanol at −78° C. All specimens were stained by the Feulgen technique before preparation of the autoradiographs.

### RESULTS

### *Effect of IPR on thymidine-5-$^3$H incorporation into nuclear DNA* and mitochondrial DNA†*

The extent of labeled thymidine incorporation into nDNA and mtDNA in the normal and host liver was similar; whereas the thymidine incorporation into tumor nDNA was twice as much as into tumor mtDNA (Table I). The specific activity in the nuclear DNA of the tumor was approximately 14 times that of specific activity of the nuclear DNA of the normal liver and host liver. The specific activity of the mitochondrial DNA of the tumor was 5–6 times the specific activity of the mitochondrial DNA of the normal liver and host liver.

The administration of 48 mg. IPR in three injections within a period of 40 hours to 150 g. rats induced a 5-fold and 8-fold increase in the incorporation of thymidine-5-$^3$H into nDNA of normal liver and host liver respectively and 2-fold increase in the mtDNA liver. On the other hand, the thymidine incorporation

TABLE I.—*Effect of Isoproterenol on Precursor Incorporation into Mitochondrial and Nuclear DNA of the Normal Liver, Host Liver and Hepatoma* 3924*A*

| | DPM/mg. DNA* | | |
|---|---|---|---|
| Thymidine-5-methyl-$^3$H incorporation | Mitochondrial fraction† | Nuclear fraction | Ratio M/N‡ |
| A. Normal Liver | | | |
| Control | 20080 | 13140 | 1·5 |
| Isoproterenol | 39560 | 65620 | 0·6 |
| B. Host Liver | | | |
| Control | 14600 | 13660 | 1·1 |
| Isoproterenol | 34020 | 104170 | 0·3 |
| C. 3924A Tumor | | | |
| Control | 98050 | 189220 | 0·5 |
| Isoproterenol | 84110 | 156520 | 0·5 |
| Cytidine-5-$^3$H incorporation | | | |
| A. Normal Liver | | | |
| Control | 16710 | 3440 | 4·9 |
| Isoproterenol | 23800 | 6950 | 3·4 |
| B. Host Liver | | | |
| Control | 16490 | 5820 | 2·8 |
| Isoproterenol | 33710 | 7750 | 4·3 |
| C. 3924A Tumor | | | |
| Control | 33800 | 26400 | 1·3 |
| Isoproterenol | 16100 | 15560 | 1·0 |

* Disintegrations per minute per milligram of DNA
† Pooled samples of 6–8 livers of tumors
‡ Mitochondrial fraction over nuclear fraction into both organelles of the hepatoma was about 85% of their control values.

All of the autoradiographic results (both grain counts per nucleus and per cent labeled cells) in the IPR treated normal liver, hepatoma and host liver were above control values. The grain counts per nucleus for the normal liver was 126% and for the host liver 124%. There was a 2·5-fold increase in the per cent labeled cells in both the normal liver and host liver of IPR treated animals (see Table III). Neither the grain counts per nucleus nor per cent labeled cells of Hepatoma 3924A were significantly elevated. They were 112% and 126% of control values respectively.

*Effect of IPR on cytidine-5-$^3$H incorporation into nuclear DNA and mitochondrial DNA*

The incorporation of cytidine-$^3$H into mitochondrial DNA of the normal liver and host liver was comparable to the incorporation of thymidine-methyl-$^3$H However, the rate of cytidine incorporation into nuclear DNA was between 1/2 and 1/5 the rate of incorporation of thymidine-methyl-$^3$H. This is shown by the ratio of the mitochondrial to nuclear DNA (M/N ratio) specific activity following cytidine and thymidine (Table I). The M/N ratio for normal liver and host liver was 1·5 and 1·1 respectively following thymidine and 4·9 and 2·8 respectively following cytidine. The specific activity of the nuclear DNA of Hepatoma 3924A following cytidine-5-$^3$H was 4 to 8 times the specific activity of the normal liver and host liver, but the mitochondrial specific activity of the tumor was only about 2 times the mitochondrial specific activity of the normal liver and host liver.

The specific activities of both nuclear and mitochondrial DNA in the normal liver and host liver of IPR treated animals were elevated (Table II). DNA specific

TABLE II.—*Effect of Isoproterenol on Nucleic Acid Labeling in Mitochondrial and Nuclear Fractions Isolated from the Normal Liver, Host Liver and Hepatoma 3924A Expressed as Per cent of Control Speciffc Activity*

| | Per cent of control specific activity* | |
|---|---|---|
| Thymidine-5-methyl-$^3$H DNA | Mitochondrial fraction | Nuclear fraction |
| Normal liver . . . . | 197 | 499 |
| Host liver . . . . | 233 | 763 |
| 3924A Tumor. . . . | 85 | 83 |
| Cytidine-5-$^3$H DNA | | |
| Normal liver . . . | 142 | 202 |
| Host liver . . . . | 204 | 133 |
| 3924A Tumor. . . . | 47 | 59 |

* Disintegration per minute per mg. DNA or RNA

activity of the normal liver and host liver were 142 and 204% of control values, respectively. The specific activities of nuclear DNA in the normal liver and host liver was 202 and 133%, respectively. There was a depression of both mitochondrial and nuclear DNA specific activity in Hepatoma 3924A after IPR administration. The values were 47 and 59% of the controls.

TABLE III.—*Autoradiographic Data*

(following thymidine-5-methyl-$^3$H administration)

| | Grain counts per nucleus | % of control | % Labeled cells | % of control |
|---|---|---|---|---|
| A. Tumor 3924A | | | | |
| Control . . | 25·6±2·2* . | | . 7·7±0·8 . | |
| Isoproterenol . | 28·9±2·0 . | 112 | . 9·7±1·8 . | 126 |
| B. Host Liver | | | | |
| Control . . | 28·0±1·7 . | | . 0·9±0·09 . | |
| Isoproterenol . | 34·8±2·5 . | 124 | . 2·2±0·6 . | 245 |
| C. Normal Liver | | | | |
| Control . . | 26·8±1·9 . | | . 0·8±0·1 | |
| Isoproterenol . | 33·6±4·1 . | 126 | . 2·0±0·4 . | 250 |

* Standard error of the mean (5–6 tumors used for each point).

### DISCUSSION

Barka (1965*a*) and Barka and Popper (1967) showed that IPR would increase significantly the number of liver cells of the rat synthesizing DNA. One single dose of IPR (160 mg./kg. body weight) resulted in a 3-fold increase in labeled thymidine incorporation into DNA (specific activity) in 24 hours. Repeated smaller doses over a 36 to 40 hour period resulted in a 7-fold increase in specific activity. Labeled cells, demonstrated by autoradiographic means, had a comparable 7-fold increase, thereby showing that the increased specific activity was primarily the result of new cells beginning DNA synthesis. IPR primarily stimulated hepatocytes to synthesize DNA since 71 to 89% of the labeled cells were parenchymal cells.

A 5-fold to 8-fold increase in specific activity in liver nDNA is comparable to Barka's finding with rat liver. The 2·5-fold increase in the per cent labeled cells in the host liver and normal liver was not as great as found by Barka The increase in the grain counts per nucleus to 124 and 126% of control values are not significant. Both grain counts per nucleus and per cent labeled cells in the IPR treated tumors were above control values; however, the " t " values did not indicate a significant elevation.

The 2-fold increase in the rate of thymidine-5-methyl-$^3H$ uptake into the mitochondrial DNA of the normal liver and host liver could be the result of an increase in rate of DNA synthesis in the mitochondria replicating DNA. IPR could result in increased numbers of mitochondria of liver cells to initiate DNA synthesis as has been demonstrated for rat liver nuclei. It would be of interest to know if the initiation for mitochondrial DNA synthesis is similar to the initiation for nuclear DNA synthesis. This question cannot be answered at the present time since it is not known how many of the estimated 800 mitochondria of the rat liver cell are in the process of DNA synthesis at any particular time (Lehninger, 1964). If it is inferred that similar mechanisms are involved in the initiation of DNA synthesis in the mitochondria and nuclei of rat liver then Barka's results and the results of this study would favor an increase in number of labeled mitochondria as the predominate mechanism for the 2-fold increase in the mitochondrial DNA specific activity in the IPR treated animals. The 245–250% increase in the number of cells initiating nDNA synthesis after IPR was greater than the 125–126% increase in the grain counts per nucleus in the host liver and normal liver.

Initial experiments of this laboratory using male Lewis strain rats produced a 1·5 to 2-fold increase in DNA specific activity. Barka (personal communication) indicated that female rats were more sensitive than males with regard to IPR stimulation of DNA synthesis in liver cells. A repeat of the experiment with female ACI rats produced the 5 to 8-fold increase reported in this study. ACI rats were used because they are routinely used for tumor transplantation. The inability of Whitlock *et al.* (1968) to repeat Barka's findings in the rat in male mouse liver may be related to either species differences between the mouse and rat or sex differences or a combination of both species and sex differences.

A greater stimulation in DNA synthesis was found in the nuclear fraction than in the mitochondrial fraction; and the effect of IPR on the incorporation of labeled cytidine was less than labeled thymidine in both organelles. Some of the possible factors which have resulted in the differences are: (1) differences in the concentration of IPR in the tumor and liver, (2) differences in the effect of IPR on the induction of enzymes involved in DNA synthesis in the resting liver and the actively proliferating tumor, (3) differences in the magnitude of precursor pool changes as a result of the effect of IPR on the cardiovascular system.

Malamud and Baserga (1967) injected labeled IPR in mice and found differences in concentration of IPR and in the total radioactivity in the liver, salivary gland, and heart. These differences could explain the discrepancy found in the stimulation of DNA synthesis among various organs. Differences in intracellular distribution and concentration of IPR could occur in the liver and tumor in this study which could be the reason for the differences in the response to IPR. Further, the concentration of catechol-o-methyl-transferase, the main catabolizing enzyme for IPR could also be different in the tumor and liver. Malamud and

Baserga (1967) and Barka (1965*a*) consider that IPR acts directly on the salivary gland cells in stimulating DNA synthesis since adrenalectomized rats were as sensitive as normal rats to IPR.

The 12 to 25% increase in the grain counts per nucleus on the liver and tumor suggests that IPR increases the rate of DNA synthesis in the liver cells already in DNA synthesis when the IPR was given. This may be related to increased blood flow through the IPR treated animal because of its vasodilitory effect on the cardiovascular system. The vasodilitory effect of IPR may also change the precursor pool size of cytidine to a greater degree than thymidine. It is known that the pool size for thymidine is small and that labeled thymidine which is not incorporated into DNA is degraded within an hour (Chang and Looney, 1965). On the other hand, with cytidine as a precursor, the appearance of labeled nucleic acids continues for several hours beyond the availability of precursor, and this increase is not stopped by flooding with unlabeled ("cold") precursor (Feinendegen *et al.* 1961). This could account for the lower rate of cytidine incorporation into nuclear DNA compared to thymidine incorporation into nuclear DNA in the liver and tumor in this and previous studies.

Whitlock *et al.* (1968) demonstrated that the increase in DNA synthesis parallels the increase in thymidine kinase activity in salivary gland after IPR. The IPR induced thymidine kinase was sensitive to low doses of Dactinomycin. Conversely, $\alpha$-amylase which is abundant in the salivary gland, varied independently of DNA synthesis and was resistant to low doses of Dactinomycin. It does mean that Dactinomycin inhibits the induction of thymidine kinase but not $\alpha$-amylase activity. Therefore, IPR may affect the initiation of the formation of messenger RNA and thus affect the genetic transcription process with regard to the production of thymidine kinase and DNA polymerase. The observed difference in the response to IPR-induced DNA synthesis resting liver and the rapidly proliferating tumor could be related to the relative effectiveness of IPR to induce the formation of enzymes actively involved in DNA synthesis in the liver and tumor.

The metabolic effects of IPR have been reviewed by Land and Browns (1967). It has been reported that catecholamines such as IPR affect the formation of cyclic 3′,5′-AMP mediated by adenyl cyclase. It has also been reported that IPR has the greatest potency of all the catecholamines on the alteration of such metabolic functions as glycolysis and lactic acid production (Sutherland and Rall, 1960). It is therefore possible that both qualitative and quantitative differences in the nucleotide pool size may result from IPR administration. Changes in nucleotide pool size and concentration could be possible mechanisms by which DNA synthesis is initiated after IPR administration since many investigators have implicated the nucleotide concentration changes in the initiation of DNA synthesis.

### SUMMARY AND CONCLUSIONS

The administration of 48 mg. isoproterenol (IPR) in three injections within a period of 40 hours to 150 g. rats induced a 5-fold to 8-fold increase in specific activity in the incorporation of thymidine-5-methyl-$^3$H into nuclear DNA and 2-fold increase in specific activity into the mitochondrial DNA of normal liver and host liver. On the other hand, the thymidine-5-methyl-$^3$H incorporation into both organelles of the hepatoma was about 85% of their control values. The grain counts per nucleus for the normal liver and host liver were 126% and 124%

respectively. There was a 2·5-fold increase in the per cent labeled cells in both the normal liver and host liver in the IPR treated animals. The grain counts per nucleus and per cent labeled cells of Hepatoma 3924A were 112% and 126% of control values respectively.

The specific activities of both nuclear and mitochondrial DNA in the normal liver and host liver of IPR treated animals were elevated following cytidine-5-$^3$H. DNA specific activity of the normal liver and host liver were 142% and 240% of control values, respectively. The specific activities of nuclear DNA in the normal liver and host liver were 202% and 133%. There was a depression of both mitochondrial and nuclear DNA specific activity in Hepatoma 3924A after IPR administration. The values were 47% and 59% of the controls.

The rate of cytidine-5-$^3$H incorporation into nuclear DNA was between 1/2 and 1/5 the rate of incorporation of thymidine-5-methyl-$^3$H. The mtDNA/nDNA specific activity ratio of the normal liver and host liver was 1·5 and 1·1 respectively following thymidine-5-methyl-$^3$H and 4·9 and 2·8 following cytidine-5-$^3$H. The specific activity of the nuclear DNA of Hepatoma 3924A following cytidine-5-$^3$H was 4 to 8 times the specific activity of the normal liver and host liver, but the mitochondrial specific activity of the tumor was only about 2 times the mitochondrial specific activity of the normal liver and host liver.

We wish to express our appreciation to Miss Linda Keyes and Mrs. Audrey Mayo for their technical assistance in this work.

The research reported herein was supported in part by U.S. Public Health Service Grants No. GM-10754, No. CA-10729 and American Cancer Society Grant No. P-497.

### REFERENCES

Barka, T.—(1965*a*) *Expl Cell Res.*, **37**, 662.—(1965*b*) *Expl Cell Res.*, **39**, 355.
Barka, T. and Popper, H.—(1967) *Medicine, Baltimore*, **46**, 103.
Baserga, R.—(1966) *Life Sci.*, **51**, 2033.
Baserga, R. and Heffler, S.—(1967) *Expl Cell Res.*, **46**, 571.
Chang, L. O. and Looney, W. B.—(1965) *Cancer Res.*, **25**, 1817.
Chang, L. O. and Looney, W. B.—(1966) *Int. J. Radiat. Biol.*, **12**, 187.
Feinendegen, L. E., Bond, V. P. and Painter, R. B.—(1961) *Expl Cell Res.*, **22**, 381.
Kirby, K. S.—(1957) *Biochem. J.*, **66**, 495.
Land, A. M. and Browns, T. G., Jr.—(1967) in 'Sympathomimetic (adrenegic) Stimulants in Drugs Affecting the Peripheral Nervous System.' Edited by A. Burger. New York (Marcel Detker, Inc.)
Lehninger, A. L.—(1964) 'The Mitochondrion'. New York (W. A. Benjamin, Inc.) p. 31.
Looney, W. B., Chang, L. O. and Banghart, F. W.—(1967) *Proc. natn. Acad. Sci. U.S.A.*, **57**, 972.
Lowry, O., Rasebrough, N., Farr, A. and Randall, R.—(1951) *J. biol. Chem.*, **193**, 265.
Malamud, D. and Baserga, R.—(1967) *Life Sci.*, **6**, 1765.
Marmur, J.—(1961) *J. molec. Biol.*, **3**, 208.
Morris, H. P.—(1965) *Adv. Cancer Res.*, **9**, 227.
Nass, S., Nass, M. M. R. and Hennix, U.—(1965) *Biochim. biophys. Acta*, **93**, 426.
Schneider, W. C. and Kuff, E. L.—(1965) *Proc. natn. Acad. Sci. U.S.A.*, **54**, 1650.
Selye, H., Veilleux, R. and Cantin, M.—(1961) *Science, N.Y.*, 133 : 44.
Sutherland, E. W. and Rall, T. W.—(1960) *Pharmac. Rev.*, **12**, 265.
Whitlock, J. P., Jr., Kaufman, R. and Baserga, R.—(1968) *Cancer Res.*, **28**, 2211.